# Health Discourse Regarding Syrian Refugees in Türkiye on X (Formerly Twitter): Longitudinal Sentiment and Stance Analysis

**DOI:** 10.2196/93227

**Published:** 2026-09-21

**Authors:** Ömer Ataç, Abdul Basit Adeel, Ibrahim Enes Ataç

**Affiliations:** 1 Department of Demography, Institute of Population and Social Research, Marmara University, Marmara Üniversitesi Göztepe Yerleşkesi, Eski İşletme Fakültesi Binası Kat:7, Kadıköy, Istanbul, 34722, Türkiye, 90 5543355456; 2 Department of Public Health, School of Medicine, Istanbul Medipol University, Beykoz, Istanbul, Türkiye; 3 Department of Sociology and Criminology, College of the Liberal Arts, Pennsylvania State University, State College, PA, United States

**Keywords:** refugees, social media, health services accessibility, natural language processing, sentiment analysis, stance detection

## Abstract

**Background:**

Since 2011, Türkiye has become the primary destination for Syrian refugees. Although health care is a fundamental human right, public discourse surrounding refugee health services can influence policy and social cohesion.

**Objective:**

The objective of our study was to examine 14 years of health-related discourse in Türkiye regarding Syrian refugees on platform X (formerly Twitter) to identify evolving patterns in sentiment, stance, and key grievances.

**Methods:**

From a dataset of 4.5 million tweets (2009-2022), 116,172 health-related posts were identified. We used a fine-tuned Turkish Bidirectional Encoder Representations from Transformers (BERT)–based large language model to perform multitask classification for sentiment, stance, and health topics. Tweets were categorized into 5 domains: provision of health care services, financing and coverage, human resources, public health and disease prevention, and access to medications and pharmaceutical services. Lift scores and heatmaps were used to analyze the relationship between keywords and public attitudes.

**Results:**

The fine-tuned Turkish BERT model achieved high classification performance, with a weighted *F*_1_-score of 0.814 for sentiment and 0.757 for stance detection. Public discourse shifted from neutral or positive tones in 2011 to increasingly negative tones over time. By 2021, negative sentiment reached 79.9% (13,148/16,456), and the anti-refugee stance peaked at 78.3% (12,882/16,456). Prominent topics evolved from provision of health care services (38/80, 47.5% in 2011) to public health and disease prevention (9428/16,456, 57.3% in 2021) and human resources (13,969/40,368, 34.6% in 2022). High lift scores revealed that an anti-refugee stance was strongly associated with keywords such as “appointment,” “vaccine,” and “free.”

**Conclusions:**

There is a marked and consistent rise in anti-refugee sentiment within Turkish digital health discourse, often fueled by misinformation and perceived systemic strain. Public health authorities should prioritize evidence-based communication strategies to counter digital polarization and ensure the legibility of health policies to the host population.

## Introduction

Since the Syrian civil war began in 2011, more than 650,000 people have died, and nearly 6 million have been forcibly displaced [1]. Among host countries, Türkiye stands out as the primary destination for Syrians, who have been granted temporary protection (TP) since April 2011. Although the number of Syrians under TP reached 3.74 million in 2021, it has declined to approximately 2.7 million as of April 2025 (3.4% of Türkiye’s population). Only 2.5% of these Syrian refugees reside in camps, while the majority live in urban and rural areas across the country [2].

Despite their temporary status, Syrians have become an integral part of Turkish society. This long-term presence poses ongoing challenges for both refugees and the local Turkish host population, particularly as they navigate shared public spaces such as neighborhoods, schools, workplaces, and health care services. Among these, access to health care is especially critical. Protecting the health of refugees is not only essential for their own well-being but also for public health more broadly. Health care provision for refugees should extend beyond emergency or curative services and include preventive care services such as vaccination, maternal care, and child health programs [2]. Türkiye has established 181 migrant health centers across various regions of the country. These centers are designed to provide some essential and preventive health services, but they cannot fulfill all health care needs [3]. For many health care needs, particularly advanced or specialized services, refugees rely on the country’s health institutions. This dependence has intensified the pressure on an already overburdened health system.

As social media use has surged globally, monitoring public opinion on these platforms has become an increasingly critical resource for addressing this gap. Social media not only reflects public attitudes but also actively shapes them. Governments, companies, and researchers rely on such platforms to track evolving public concerns and conduct real-time policy analysis, such as public health agencies tracking vaccine hesitancy or researchers monitoring infodemics during global health crises [4,5]. Yet the same features that make social media a valuable tool for monitoring public opinion also render it vulnerable to manipulation and misinformation [5]. The rapid dissemination of unverified or misleading content can distort public discourse and deepen polarization. In Türkiye, where social media use is particularly widespread, understanding digital conversations is especially relevant for capturing shifts in public attitudes and helping policymakers develop better-informed policies [6].

To systematically analyze public attitudes on social media, computational techniques such as *sentiment analysis* and *stance detection* have gained prominence. Sentiment analysis involves the classification of textual content based on its emotional tone, typically into positive, negative, or neutral categories [7]. Although this method is helpful for capturing the general valence of a message, it does not specify the target of that emotion or the position taken toward it [8]. To further strengthen the analysis and provide a more comprehensive assessment of public opinion, we complement sentiment analysis with stance detection. Stance detection, while related, differs in that it estimates the author’s position toward a specific topic or group, even when that target is not explicitly mentioned in the text [9]. It classifies text into predefined categories such as pro, against, or neutral, thus providing a more targeted understanding of opinion [10]. This distinction is particularly relevant for refugee health in Türkiye, where expressions of concern, frustration, or criticism may be directed at institutions, policies, or resource allocation rather than at refugees themselves.

Beyond its policy implications, refugee-related health care is a deeply social issue shaped by public grievances and group dynamics. Despite growing digital public health research, no study has systematically analyzed longitudinal health-related discourse regarding Syrian refugees on Turkish social media. This study addressed this gap by analyzing health-related Turkish tweets about Syrian refugees on platform X (formerly Twitter) from 2009 to 2022. Using large-scale sentiment and stance analysis, we investigated temporal trends and key shifts in public opinion, providing insights to inform more responsive and equitable health care strategies.

## Methods

### Data Source and Study Design

In this study, we retrieved approximately 4.5 million publicly available tweets posted between 2009 and 2022 by querying the Turkish keywords *suriyeli* and *suriyeliler* (“Syrian” and “Syrians,” respectively) using Python’s *snscrape* library (version 3.13; Python Software Foundation) in June 2023. Because X substantially restricted API access in 2023, we were unable to extend data collection to later years. Consequently, the dataset does not capture posts from 2023 onward, including reactions to major events such as the 2023 earthquakes, which may have further influenced public attitudes toward Syrian refugees. We retained 3 million of these refugee-related tweets after removing duplicate tweets, retweets, and tweets containing only links. For this paper, our analysis followed a 2-step process: first, we identified all health-related tweets among these 3 million tweets, and second, within this medical subset, we classified each tweet according to its sentiment, stance, and health-related topics and subtopics.

To accomplish the first step, we randomly drew a training set of 26,000 tweets using year-stratified sampling to ensure its representativeness. The tweets were then manually annotated by our coders, including 1 public health professional (ÖA) and 1 coder with a social science research background (IEA), both native Turkish speakers. This training set was used to train a machine learning classifier that identified our final analytical sample of 116,172 health-related tweets. For the second step, we annotated subsets of these tweets to create training data for the remaining classification tasks (approximately 10,000 tweets each for sentiment, stance, and subtopics, and 26,000 for main topics). We stopped annotating tweets for training purposes when our model’s evaluation metrics reached sufficient levels (discussed below).

Health topics were defined using the World Health Organization (WHO) health systems framework as a conceptual guide and further refined based on the European Observatory on Health Systems and Policies Health in Transition (HiT) Series [11,12]. We developed five main topics and their corresponding subtopics to classify the health-related tweets:

1. Provision of health care services: priority access, overcrowding, and general health care services
2. Financing and coverage: free health care services, health care costs and fees, and health insurance
3. Human resources: health care workforce and health care students
4. Public health and disease prevention: threat of new diseases or outbreaks, vaccination and immunization, screening, COVID-19, health problems, and fertility
5. Access to medication and pharmaceutical services: medications, prescriptions, and pharmacy

Subtopics were derived inductively during manual annotation of 26,000 tweets, based on recurring themes in public discussions. The final categorization remained conceptually aligned with the cited policy documents.

Our team of coders achieved an intercoder reliability of 97.4% agreement (Cohen κ=0.95) for sentiment and 91.5% agreement (Cohen κ=0.84) for stance, based on an initial set of 3000 tweets (11.5% of the 26,000-tweet medical relevance set and 30% of the 10,000-tweet sentiment and stance sets). A Cohen κ value >0.80 is considered near-perfect agreement and is widely accepted as the threshold for reliable annotation in social media research [13,14].

### Outcome Measures and Data Analysis

Our primary outcomes were the trends and distributions of the number of health-related tweets over time; the main topics and subtopics discussed each year; the sentiment expressed in each tweet, capturing its overall emotional tone and coded as positive, negative, or neutral based on the valence of the text; and the stance expressed in each tweet, capturing the position taken toward Syrian refugees in the context of health and coded as pro, anti, or neutral.

Turkish-language natural language processing (NLP), particularly for informal X data, remains less well-resourced than English-language NLP, which introduces important limitations for classification tasks. Turkish is an agglutinative language with rich morphology, and tweets frequently contain nonstandard spelling, slang, abbreviations, missing diacritics, hashtags, and context-dependent expressions such as irony or sarcasm, all of which can reduce the reliability of off-the-shelf models [15]. These challenges are especially relevant for more interpretive labels such as sentiment and stance and may have contributed to lower performance in those categories relative to more concrete labels such as medical relevance or topic classification. To mitigate these limitations, we fine-tuned a Turkish Bidirectional Encoder Representations from Transformers (BERT; Alphabet Inc)–based model on our annotated dataset to classify each tweet across 5 related labels, namely, medical relevance, topic, subtopic, sentiment, and stance. We further report label-specific performance metrics in the evaluation results to make the quantitative impact of these challenges transparent [16].

We used the pretrained Turkish BERT base model (*emrecan/bert-base-turkish-cased-mean-nli-stsb-tr*). Because it was pretrained on a large corpus of Turkish text, it already understands core Turkish grammar, word forms, and common use patterns, which helps the model interpret the informal and colloquial language typical of social media. We fine-tuned this BERT encoder on our annotated tweet dataset and attached 5 task-specific classification heads (1 per task) so that a single shared language representation could support multiple outputs (medical relevance, topic, subtopic, sentiment, and stance). This multitask setup is beneficial because training on a higher-resource task can improve performance on related lower-resource tasks via inductive transfer [17]. In our data, medical relevance had approximately 26,000 labeled tweets available, whereas tasks such as stance and sentiment had approximately 10,000 labeled tweets available. In addition, not every tweet in the training set was annotated for every task; therefore, during training, we computed the loss only for the tasks that had a ground-truth label for a given tweet, allowing us to use partially labeled examples without discarding them.

For evaluation, we used stratified k-fold cross-validation to make results more robust and less dependent on a single train-test split. We split the data into 30 folds for the labeling task (and 25 folds for the medical relevance classifier), trained the model separately for each fold, and used each fold once for validation while keeping label proportions consistent across folds (eg, keeping “pro, anti, and neutral” distributions similar). To reduce overfitting, we used a random dropout rate of 0.5 and an early stopping criterion, stopping training if validation performance did not improve for 3 consecutive epochs. We focused on minimizing cross-entropy loss during training and selected the final reported model based on the best average weighted *F*_1_-score across validation folds. Weighted *F*_1_-score is well suited here because social media labels are often imbalanced, and it provides a balanced view of performance across classes.

To analyze associations between medical-related keywords and discourse outcomes (sentiment, stance, and topics), we used lift scores in addition to raw frequency counts. Lift is a well-established measure in data mining and association rule learning that quantifies the degree to which a feature’s occurrence in a target group differs from statistical independence [18]. In our implementation, we operationalized lift as the ratio of the percentage of a word’s occurrences within a sentiment category to the percentage of tweets in that sentiment category, indicated as follows: Lift (word, sentiment) = proportion of word in sentiment / proportion of tweets in sentiment

Raw frequency counts fail to account for distributional imbalances. For instance, if positive tweets constitute 60% of the corpus and negative tweets constitute 20%, a word such as “doctor’’ appearing 600 times in positive tweets and 200 times in negative tweets would show identical proportional use, yet raw frequencies would misleadingly suggest a 3-fold positive preference.

Lift scores normalize for base-rate differences. A lift of 1.0 indicates that a word appears at the expected rate (no association); a lift >1.0 indicates a positive association (the word is characteristic of that sentiment); and a lift <1.0 indicates a negative association (the word is avoided). For example, a lift of 2.5 means a word appears 2.5 times more frequently in a sentiment than expected under independence. Raw frequencies often obscure such semantic associations. This normalization is particularly valuable in textual analysis with class imbalance, as in our dataset, and it helps identify genuinely distinctive words rather than merely frequent ones. To illustrate these patterns more clearly, we selected example words from the top 50 terms ranked by lift scores within each sentiment class.

We conducted descriptive analyses to examine trends in health-related discourse across years. We calculated yearly frequencies and proportions for each health topic, subtopic, sentiment, and stance category. Temporal changes were visualized using line graphs and tables. Subtopic-level shifts were examined within each main topic to identify emerging themes. All analyses and visualizations were conducted using Python.

### Ethical Considerations

This study analyzed secondary, publicly available posts from the social media platform X related to refugees. Data collection and analysis were conducted strictly via the official X API in compliance with the platform’s terms of service and established ethical guidelines for social media research. To protect user privacy, the collected data were limited to public tweet texts and generic metadata; no usernames, private profile information, or other directly identifiable data were collected, stored, or reported. All metrics (sentiment, stance, and topics) were computed at the aggregate level, and any representative tweets are presented in paraphrased form to ensure that no individual-level data can be traced back to specific accounts. The raw dataset is stored securely and is accessible only to the research team.

Under Pennsylvania State University policy RP03, “research involving human participants” requires active intervention or interaction or obtaining identifiable private information [19]. Because our study relied entirely on public posts (where users have no expectation of privacy), involved no interaction with individuals, and collected no private data, it does not constitute human subjects research. As policy RP03 specifies that activities falling outside this definition do not require institutional review board (IRB) submission, review, or approval, this study was deemed exempt, and informed consent was not required.

## Results

Our classifiers achieved strong performance in identifying and categorizing health-related discourse. Model performance was evaluated using *k*-fold cross-validation and is reported in Table 1 using accuracy, weighted *F*_1_-score, and macro *F*_1_-score. Accuracy indicates how often the model assigned the correct label overall. *F*_1_-score provides a balanced summary of classification performance across classes. Because several tasks involved multiple categories with uneven numbers of examples, weighted *F*_1_-score is reported as the primary summary metric, while macro *F*_1_-score is included to show performance across classes with equal weight.

**Table 1.** Performance metrics for multitask classification models used to identify medical relevance, topics, sentiment, and stance in tweets.

Classifiers	Labels, n	Accuracy	Weighted *F*_1_-score	Macro *F*_1_-score	Difference (accuracy–weighted *F*_1_-score)
Medical relevance	2	0.896	0.894	0.889	0.002
Main topic	6	0.845	0.846	0.809	–0.001
Subtopic	18	0.803	0.803	0.682	~0.000
Sentiment	3	0.818	0.814	0.746	0.004
Stance	3	0.758	0.757	0.715	0.001

**Figure 1.**
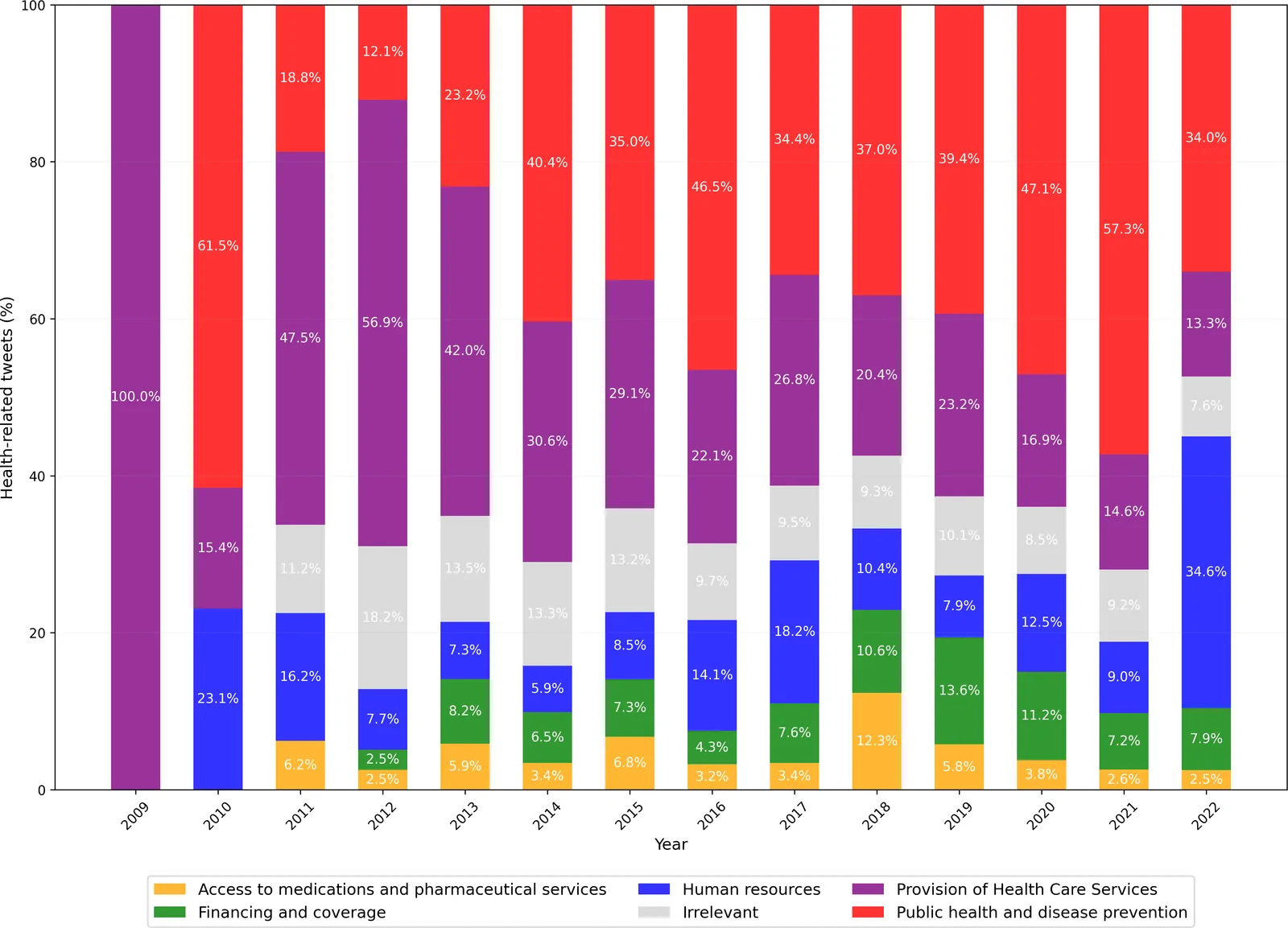
Distribution of main topics in health-related tweets by year (%).

Among our sample of 116,172 health-related tweets, the focus of health-related discussions shifted over the years (Figure 1). Initially, conversations centered on the provision of health care services, which accounted for 47.5% (38/80) and 56.9% (781/1373) of tweets in 2011 and 2012, respectively. However, from 2014 onward, public health and disease prevention became the dominant topic, peaking at 57.3% (9428/16,456) of the discourse in 2021. In 2022, there was a dramatic surge in discussions about human resources, which comprised 34.6% (13,969/40,368) of all health-related tweets that year, up from just 9% in the year before.

Within these topics, specific subtopics also experienced major shifts (Figure S1 in Multimedia Appendix 1). For instance, discussions around fertility became a prominent subtopic, accounting for >30% of the subtopic discourse in some years. Similarly, the COVID-19 pandemic appeared as one of the most prominent subtopics in 2020 and 2021, representing 16.1% (2060/12,792) and 25.9% (4262/16,456) of subtopic discussions in those years, respectively.

**Figure 2.**
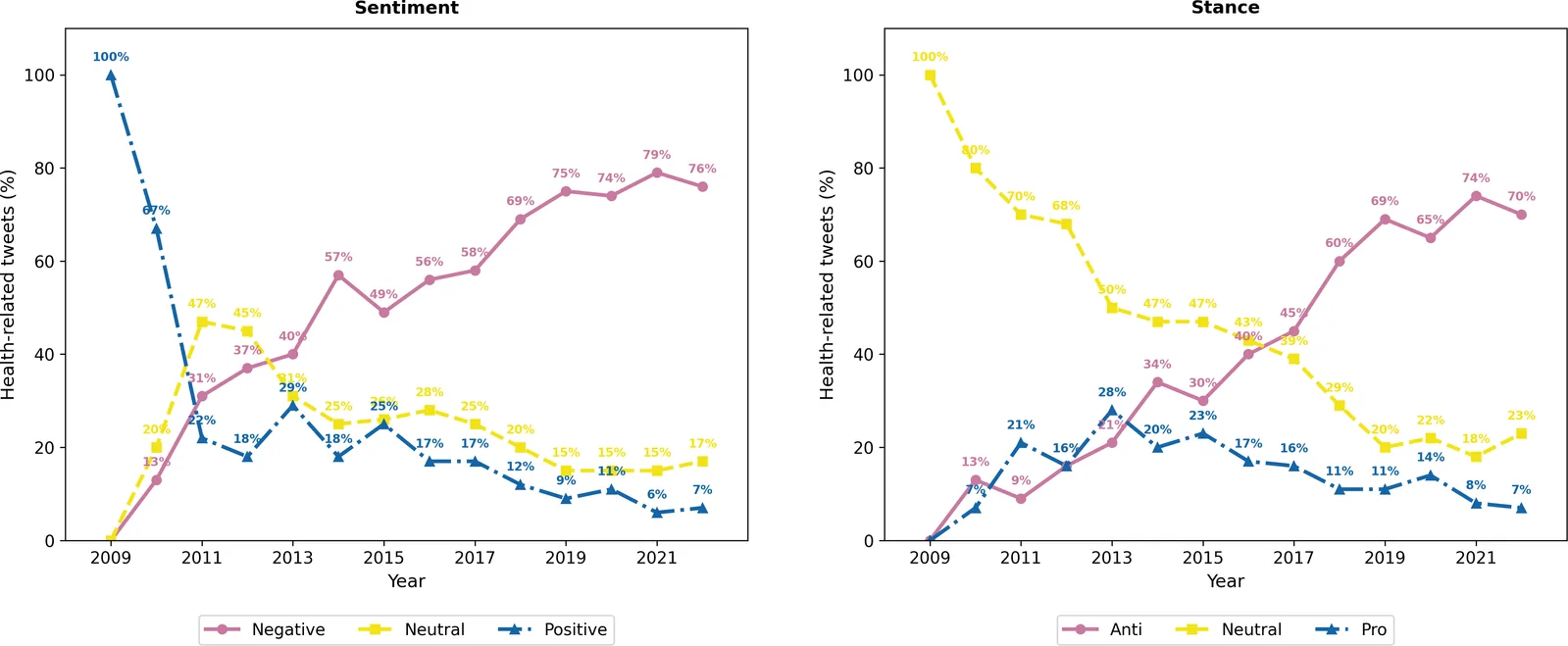
Annual distribution of sentiment and stance in health-related tweets about Syrian refugees in Türkiye (2009-2022, %).

The emotional tone and underlying stance of the health discourse became markedly more negative and anti-refugee over time (Figure 2). In the early years, the sentiment was largely positive or neutral. However, a negative shift began around 2013, and negative sentiment consistently dominated the conversation, comprising >70% of tweets in most years and peaking at 79.9% (13,148/16,456) in 2021. The pattern for stance was even more pronounced. In 2013, the discourse was balanced, with pro-refugee (706/2945, 24%) and anti-refugee (810/2945, 27.5%) stances accompanying a neutral majority. By 2017, this had inverted, with an anti-refugee stance making up 50.1% (3290/6567) of tweets. This anti-refugee sentiment remained the dominant stance in subsequent years, reaching 78.3% (12,882/16,456) in 2021. In each year since 2018, the proportion expressing an anti-refugee stance has been nearly twice as high as the combined proportions expressing pro-refugee and neutral stances.

According to the sentiment heatmap, general health terms such as “health,” “doctor,” or “patient” remain emotionally neutral or positive across the discourse (Figure S2 in Multimedia Appendix 1). However, words associated with health systems, such as “precaution,” “restriction,” “system,” “hospital,” and “economy,” concentrate in negative sentiment. Similar patterns were observed in the stance heatmap (Figure S3 in Multimedia Appendix 1). Extreme lift scores (>8.0) were observed for “medicine” in the medication subtopic, “vaccine” in the vaccination-immunization and COVID-19 subtopics, “mask” in the COVID-19 subtopic, and “appointment” in the priority access subtopic (Figure S4 in Multimedia Appendix 1).

We also compared health-related discourse with the general discourse regarding Syrian refugees (Table S1 in Multimedia Appendix 1). The results show that health discourse is moderately more negative (4.3 pp) and more anti-refugee (7.4 pp) than general refugee discourse.

## Discussion

### Principal Findings

In this study, we examined 14 years of public discourse on X regarding Syrian refugees and health in Türkiye. Our results suggest a marked and consistent rise in both negative sentiment and anti-refugee stance over time. Recently, nearly three-quarters of health-related tweets reflected negative attitudes, while positive or pro-refugee expressions fell below 10%. These findings strongly indicate that public attitudes toward Syrian refugees have become increasingly negative over time.

To our knowledge, this is the first study to examine long-term sentiment and stance trends around refugee health on Turkish social media. The rise in negative discourse closely mirrors broader public opinion trends in national surveys. Most notably, the Syrians Barometer has consistently reported a decline in public acceptance of Syrians since 2017, with the 2023 wave reporting record-high support for refugee return (89%) and forced deportation (73%). Key drivers of this trend include the scale and perceived permanence of displacement, economic hardships exacerbated by rising domestic inflation, and the growing politicization of refugee-related issues across traditional and digital media platforms, which frequently intensify during political election cycles [20].

The scope of services provided to refugees remains a widely debated issue. However, health is a fundamental human right, affirmed by numerous international legal documents that apply universally, regardless of citizenship or legal status [21-24]. These frameworks also define host countries’ responsibilities for providing health care to displaced populations [25]. Refugees fleeing war often escape under extremely harsh and traumatic conditions, exposing them to elevated health risks. The experience of displacement, marked by exposure to violence, psychological trauma, and inadequate access to basic needs such as food, clean water, and hygiene, can severely compromise their well-being [26-30].

In Türkiye, Syrian refugees’ access to health care is regulated by specific legal frameworks. Under TP, refugees are eligible to benefit from most health services. However, legal and structural barriers continue to hinder effective access, including bureaucratic limitations, physical inaccessibility, health care professional shortages, and language barriers [30]. Nevertheless, a survey showed that health care remains the most positively rated public service among Syrians, with >60% reporting no access issues in most years since 2017, except during the pandemic. In the same study, only 2.5% identified health care as a main problem, placing it at the bottom of all listed concerns [20].

One of the most frequently criticized issues in public discourse in Türkiye is government spending on Syrian refugees, particularly spending on health care. In our study, these criticisms are especially evident in subtopics such as free health care services, medications, and pharmacy, where users frequently express frustration that Syrians receive services at no cost. Similar attitudes have been reported in other studies, noting the perceived injustice of Syrian refugees receiving free health care while Turkish citizens are required to pay copayments and examination fees [30]. However, health expenditures for Syrians in Türkiye are primarily funded through the Presidency of Migration Management under the Ministry of Interior, and the European Union (EU)–funded SIHHAT (Supporting Migrant Health Services in Turkey) project. Expenditures by the Presidency of Migration Management follow a universal health coverage approach aligned with Türkiye’s Social Insurance and Universal Health Insurance Law (Law number 5510). Article 60 of this law ensures that refugees and stateless persons have access to the general health insurance system in Türkiye. In 2024, the Directorate General of International Protection had an allocated budget of approximately ₺10 billion (₺1=US $0.0305, as per the average 2024 exchange rate), with the majority of this budget allocated to health-related expenditures [31]. Our findings indicate that despite these established institutional funding mechanisms, public resentment regarding the financial burden has intensified alongside rising living costs, making citizens more sensitive to public spending on refugee health care.

Launched in 2015 through a partnership between the EU and the Turkish Ministry of Health, the SIHHAT project has significantly expanded health care services for refugees in Türkiye [32]. Funded with €210 million (€1=US $1.1096, as per the average 2015 exchange rate) under the EU Facility for Refugees in Türkiye (FRIT), the project has supported the establishment of migrant health centers, recruited health care personnel, procured medical equipment for primary and secondary care facilities, and provided vaccines. As of 2021, 181 migrant health centers and 10 community mental health centers were operating in 29 provinces with high refugee populations. These centers employ nearly 750 physicians and 4000 health care workers, mostly Syrian nationals. By the end of 2021, the project facilitated more than 93 million medical consultations and emergency services for more than 930,000 individuals. Additionally, more than 8.6 million vaccine doses were administered between 2014 and 2021 [32].

Public complaints regarding Syrian refugees’ access to health care in Türkiye often focus on hospital overcrowding and appointment shortages, frequently attributed to the refugee population. Some posts claim that Syrians receive appointment priority. Türkiye ranks second among Organisation for Economic Co-operation and Development (OECD) countries in physician visits per capita (11.4), and appointment shortages, particularly in tertiary public hospitals in major cities, are a real challenge [33]. Still, many of these grievances likely stem from broader systemic strain, with refugees serving as convenient scapegoats.

Discourse themes shifted over time. Early discussions focused on service provision, followed by an emphasis on public health and disease prevention by 2014. By 2022, however, the conversation shifted toward human resources, reflecting a heightened public focus on health care worker retention and the workload burden within public hospitals. Alongside these structural concerns, “fertility” remained a consistent subtheme, with criticism directed at perceived high birth rates. Since 2011, nearly 900,000 Syrian children have been born in Türkiye, and the fertility rate among Syrians (5.3) in Türkiye exceeds the national average [1,34]. However, studies suggest this is not a continuation of prewar Syrian trends but rather a consequence of delayed marriages and postponed childbirth and is not expected to persist long-term [35].

As reflected in our results, social media discourse on Syrian refugees and health is largely dominated by misinformation. For instance, claims of preferential health care access are incorrect, as service prioritization follows Ministry of Health protocols rather than nationality [36]. Similarly, allegations linking refugees to widespread communicable disease outbreaks lack epidemiological support. Although some localized increases occurred early in the displacement period, tuberculosis prevalence among refugees was comparable to national rates, malaria was absent, and measles outbreaks were reversed through mass immunization efforts [37,38]. Potential polio risks were likewise mitigated through vaccination, and trends in cutaneous leishmaniasis reflected broader regional patterns rather than refugee-specific effects [39]. During the COVID-19 pandemic, some narratives, often promoted by pandemic denialists, falsely claimed that Syrians were not contracting the virus. In reality, Syrians did contract COVID-19 and received the same treatment as citizens, although challenges in case detection and citizenship-based reporting may have led to underrepresentation in official statistics.

Another topic concerns the employment of Syrian health care workers. Misconceptions include claims that Syrians work in public hospitals or receive medical school placements without exams. In reality, Syrian health care workers, including physicians, are exclusively employed in migrant health centers and authorized to provide services to Syrians under TP. Similarly, Syrians studying medicine in Türkiye must either pass the foreign student entrance exam or fulfill eligibility requirements for transfer, like other international students [40].

Our sentiment heatmaps showed that terms such as “hospital,” “system,” and “ban” are strongly associated with negative sentiment, whereas “health,” “treatment,” and “baby” were associated with positivity. Stance heatmaps revealed starker divides: words such as “vaccine,” “appointment,” and “citizen” correlated with anti-refugee stances, while “child” and “treatment” appeared in pro-refugee posts. Notably, the term “citizen” emerged as an ideological marker in debates over entitlement. Subtopic-specific vocabulary (eg, “baby” and “birth” in fertility subtopic) points to highly segmented, policy-specific framings of refugee health.

Together, our findings point to a broader problem of policy legibility, reflecting the state’s limited capacity to observe and interpret public concerns surrounding refugee health. This challenge is particularly acute in Türkiye, where systematic public opinion surveys on health care, especially those capturing grievances related to refugees, are scarce. In the absence of such data, policymakers may lack an accurate understanding of societal tensions, allowing misinformation and polarized narratives to fill the gap. Addressing this limitation, our study demonstrates that social media discourse can serve as a complementary data stream that captures public concerns at a granularity and frequency that traditional surveys cannot match. The topic-based analysis reveals how negative sentiment attaches to different policy domains over time, shifting from service provision and overcrowding in the early years to human resources, free health care services, and fertility in later years, allowing policymakers to identify not just that the public is dissatisfied, but what they are dissatisfied with and when each concern peaks. By linking discourse patterns directly to WHO health system building blocks (eg, vaccination and immunization and human resources), the approach grounds social media analysis in the service categories that health ministries actually manage, supporting evidence-based resource allocation, risk communication planning, and early detection of emerging public concerns without relying on partisan or electoral event markers.

### Strengths and Limitations

This study represents the first systematic analysis of social media discourse regarding health among Syrians in Türkiye. Drawing on a large sample, it goes beyond traditional sentiment analysis by incorporating stance detection to better capture the nuances of online engagement. By categorizing topics and subtopics, it also identifies the dominant themes and narratives shaping the debate. We hope our findings can inform policy responses and help reduce public misunderstanding about the impact of Syrian refugees on the health care system.

This study has several limitations. First, it relies on social media data, which may not fully represent the broader population and is susceptible to user biases or manipulation. Second, Turkish NLP is still a developing field, with fewer mature resources and pretrained models than English and other high-resource languages. We addressed this by fine-tuning a Turkish BERT model on our annotated dataset rather than relying on a ready-made classifier trained on general sentiment or stance detection tasks. Fine-tuning allows the model to learn the specific linguistic patterns, colloquial expressions, and domain vocabulary of Turkish health care discourse on social media, substantially improving classification accuracy over off-the-shelf alternatives. Nevertheless, the model may still struggle with sarcasm, mixed sentiments, or culturally specific expressions that deviate from standard sentiment patterns (refer to Multimedia Appendix 1 for a detailed analysis and representative examples of these linguistic challenges).

Third, social media discourse is often reactive to short-term events (eg, elections, border incidents, and economic downturns), which may temporarily skew sentiment and stance trends without reflecting policy or service changes. Fourth, X users self-select when and whether to engage with refugee-related topics, potentially leading to underrepresentation of sensitive issues and obscuring shifts in attitudes among disengaged users. Future research should integrate social media analyses with survey data to address these limitations. Additionally, although we identify specific misinformation narratives circulating in the corpus, the study design does not allow the causal decomposition of individual stances into misinformation-driven vs grievance-driven components, as this would require knowledge of each author’s prior attitudes and information exposure history that is not recoverable from tweet text alone. Future research combining social media analysis with individual-level survey or panel data could better disentangle these mechanisms. Finally, the dataset extends through 2022, as large-scale data collection via the Academic Research API for X became prohibitively inaccessible following platform ownership changes in early 2023. Consequently, significant events such as the 2023 earthquake and general election fall outside the scope of this analysis.

### Conclusions

This study underscores the growing negativity in online public discourse surrounding Syrian refugees’ access to health care in Türkiye. Although health care remains the most positively rated service among refugees, online narratives are increasingly shaped by misinformation, selective perceptions, and political instrumentalization. These dynamics distort public understanding of refugees’ actual health care use and entitlements and risk steering policy responses toward emotionally driven rather than evidence-based decisions. As such, targeted efforts to counter misinformation and strengthen evidence-based communication in digital spaces are essential for fostering a more balanced and inclusive public dialogue. Our findings from social media offer a data-driven foundation to help address information gaps in health care policy, particularly in settings where traditional public opinion data are limited or absent.

## Supplementary material

10.2196/93227 Multimedia Appendix 1 Analysis of sarcasm and figurative language in Turkish tweets, a comparison of health-related with general refugee discourse, and supplementary figures on subtopic distribution and keyword lift scores.

## Abbreviations

BERT: Bidirectional Encoder Representations from Transformers
EU: European Union
FRIT: Facility for Refugees in Türkiye
HiT: Health in Transition
IRB: institutional review board
NLP: natural language processing
OECD: Organisation for Economic Co-operation and Development
SIHHAT: Supporting Migrant Health Services in Turkey
TP: temporary protection
WHO: World Health Organization
