# Supplementary material for: Health Discourse Regarding Syrian Refugees in Türkiye on X (Formerly Twitter): Longitudinal Sentiment and Stance Analysis
Source: J Med Internet Res. 2026 Sep 21;28:e93227. doi: 10.2196/93227 (PMC13592665; doi:10.2196/93227)
Supplement: Supplementary file 1 [file jmir-v28-e93227-s001.docx]

**Analysis of Sarcasm, Irony, and Figurative Language in Turkish Digital Discourse**

Turkish social media is rich in sarcastic and ironic expression. To quantify this, we analyzed disagreements between our two native Turkish coders in the inter-coder reliability set of 3,000 tweets. Human coders disagreed on 2.6% of sentiment annotations (77/3,000) and 8.5% of stance annotations (256/3,000). A substantial proportion of these disagreements involve sarcastic, ironic, or hyperbolic language. This establishes an irreducible error through cases where even native Turkish speakers cannot agree on the author's intent.

The model's cross-validation error rates (approximately 18% for sentiment and 24% for stance in Table 1) exceed these human disagreement rates, indicating that tweets involve figurative language that both coders interpreted literally and agreed upon, yet the model still struggles with classifying them correctly. This is the linguistic nuance of Turkish language, which establishes a ceiling effect even with the carefully annotated and strictly trained models.

Below are representative examples from our dataset where sarcasm or irony led to coder disagreement:

**Example 1**

*Turkish:* "mesela çok pis suriyeli sever"

*English:* "For example, I really *love* Syrians a lot"

*Coding:* Coder 1 = Positive, Coder 2 = Negative

"Çok pis sever" (literally "love very badly") is a Turkish colloquial intensifier that, in this context, is clearly ironic - the speaker offers no genuine justification for the claimed affection. A surface-level sentiment model trained on word valence would capture only the positive words.

**Example 2**

*Turkish:* "Suriyeli hastanede nargile içiyor, vatandaş özel alanında tek başına sigara içemiyor."

*English:* "A Syrian smokes nargile in the hospital, while a citizen cannot smoke alone in a private area."

*Coding:* Coder 1 = Neutral, Coder 2 = Negative

The juxtaposition of two scenarios criticizes perceived double standards in healthcare access, but the sentence contains no explicit negative sentiment markers i.e., no profanity, anger words, or direct accusations. The negativity is entirely carried by the contrast itself.

**Example 3**

*Turkish:* "suriyeliler oluyor da bu bebe neden olmuyor"

*English:* "Syrians are allowed, so why isn't this baby allowed?"

*Coding:* Coder 1 = Neutral, Coder 2 = Negative

A rhetorical question implying Syrians receive preferential treatment. The surface form is grammatically neutral, but the pragmatic intent is a complaint. Turkish social media frequently uses this rhetorical-question-as-criticism pattern.

**Example 4**

*Turkish:* "Ulan sahtekarlar birleşmiş milletler kürsüsünde elimiz güçlü bir şekilde bağırdıysak aramızdaki suriyeliler sayesinde olmuştur."

*English:* "You frauds, if we shouted strongly at the UN podium, it was thanks to the Syrians among us."

*Coding:* Coder 1 = Anti, Coder 2 = Pro

Mock gratitude - the speaker attributes Türkiye's diplomatic standing to Syrians as a way of ridiculing the idea. One coder read it as anti-refugee (the intended sarcasm), the other as pro-refugee (the literal meaning). This is a classic sarcasm failure mode for automated classifiers: the literal and intended meanings are opposites.

**Example 5**

*Turkish:* "hadi destek verin suriyeli kardeslerinize"

*English:* "Come on, support your Syrian brothers"

*Coding:* Coder 1 = Positive, Coder 2 = Negative

A directive that sounds supportive on the surface but is delivered sarcastically to criticize perceived favoritism. Without tone-of-voice or situational context, a classifier cannot distinguish genuine advocacy from mockery.

There are sociolinguistic variations that confuse even the most attentive human coder, but the model performs relatively well within the acceptable margins of error even under these challenges and linguistic complexities.

**Table S1**. Comparison of Sentiment and Stance Distributions Between Health-Related and Other Refugee Discourse on X

| **Metric** | **Health-related (n=116,172)** | **Others (n=2,850,192)** | **Difference** |
| --- | --- | --- | --- |
| Sentiment Negative | 72.2% | 67.9% | +4.3 pp |
| Sentiment Neutral | 19.2% | 22.5% | -3.3 pp |
| Sentiment Positive | 8.6% | 9.7% | -1.1 pp |
| Stance Anti | 66.3% | 58.9% | +7.4 pp |
| Stance Neutral | 24.5% | 27.7% | -3.2 pp |
| Stance Pro | 9.3% | 13.4% | -4.1 pp |

Figure S1: Distribution of Subtopics in Health-Related Tweets by Year (%)

Figure S2: Heatmap of lift scores for the selected key terms across sentiment categories

Figure S3: Heatmap of lift scores for the selected key terms across stance categories

Figure S4: Heatmap of lift scores for the selected key terms across subtopics
